# Healthcare-Associated Infections in Deceased Stroke Patients in a Romanian Neurological ICU: A Retrospective Descriptive Study

**DOI:** 10.3390/microorganisms14051062

**Published:** 2026-05-08

**Authors:** Simona Ioana Adriana Mlendea (Gălbineanu), Alin Kraft, Cristian Falup-Pecurariu, Tatiana Gianina Melicianu, Laurențiu Dănuț Nedelcu

**Affiliations:** 1 Department of Anaesthesia and Intensive Care, Brașov County Emergency Clinical Hospital, 500326 Brașov, Romania; sgalbineanu@gmail.com; 2 Doctoral School in Medicine, “Transilvania” University of Brașov, 500019 Brașov, Romania; laurnedelcu@yahoo.com; 3 Department of Medical-Surgical and Prophylactic Disciplines, Faculty of Medicine, Titu Maiorescu University of Bucharest, 031593 Bucharest, Romania; 4 Department of General Surgery, “General Doctor Aviator Victor Anastasiu” National Aeronautical and Space Medicine Institute, 010242 Bucharest, Romania; 5 Department of Neurology, Brașov County Emergency Clinical Hospital, 500326 Brașov, Romania; crisfp100@yahoo.com; 6 Faculty of Medicine, “Transilvania” University of Brașov, 500019 Brașov, Romania; 7 Department of Infectious Diseases, Brașov County Emergency Clinical Hospital, 500326 Brașov, Romania; dr.melicianutatiana@yahoo.com

**Keywords:** healthcare-associated infections, stroke-associated pneumonia, urinary tract infection, neurocritical care, neurological intensive care unit, infection documentation, antimicrobial resistance, Romania

## Abstract

Healthcare-associated infections (HAIs) are clinically relevant complications in critically ill stroke patients, particularly in neurological intensive care settings, where severe neurological injury, dysphagia, immobilization, invasive device exposure, and prolonged hospitalization increase infection susceptibility. Romanian data focused on deceased stroke patients admitted to neurological intensive care units remain limited. This retrospective descriptive single-center hospital-based study, supported by focused literature contextualization, was conducted in the Neurological Intensive Care Unit of the Brașov County Emergency Clinical Hospital, Romania. Adult stroke patients who died during hospitalization over a six-year observation period were included. Clinical data were extracted from a working hospital database and analyzed descriptively after data cleaning and harmonization. The final cohort comprised 190 deceased stroke patients; ischemic stroke was documented in 69.5% and hemorrhagic stroke in 28.9%. Hypertension (73.7%) and ischemic heart disease and/or previous myocardial infarction (60.0%) were the most frequently recorded comorbidities. Pneumonia was the dominant documented infectious complication, recorded in 52.6% of patients, followed by urinary tract infection (11.6%), pressure sore-related infection (4.7%), and sepsis-related coding (6.8%). The median in-hospital survival interval was 6 days (IQR 3.0–10.75). Because year-by-year stratification was not sufficiently robust, the temporal component was interpreted only in aggregate form. These findings provide a descriptive hospital-based profile of documented infectious complications in a fatal stroke ICU cohort and support the need for more standardized infection documentation and better linkage between clinical and microbiological data in neurocritical care settings.

## 1. Introduction

Healthcare-associated infections (HAIs) remain a major challenge in critically ill hospital populations, particularly in intensive care settings where severe illness, invasive device exposure, antimicrobial pressure, and prolonged hospitalization converge. In neurological intensive care units, this problem is especially relevant because patients with severe stroke are frequently exposed to impaired consciousness, dysphagia, aspiration risk, immobilization, urinary catheterization, mechanical ventilation, vascular access devices, and extended hospital stays. Against this background, HAIs in severe stroke patients represent an important topic at the intersection of neurocritical care, hospital epidemiology, infection prevention, and public health microbiology.

Stroke remains one of the leading causes of death and long-term disability worldwide and continues to generate a substantial burden in terms of mortality, disability-adjusted life years, and healthcare utilization. The most recent Global Burden of Disease analysis confirmed that, although age-standardized rates have changed over time, the absolute burden of stroke increased markedly between 1990 and 2021, underlining the persistent importance of stroke as a major public health challenge [1]. In critically ill stroke patients, prognosis is not determined solely by the primary neurological insult, but also by systemic complications acquired during hospitalization, among which HAIs are particularly consequential. Infectious complications after acute stroke have repeatedly been associated with worse prognosis. In the landmark meta-analysis by Westendorp et al., infection complicated approximately 30% of acute stroke cases, while both pneumonia and urinary tract infection occurred in around 10% of patients; notably, pneumonia was associated with increased mortality [2]. More recent evidence confirms that post-stroke infections remain a clinically relevant problem despite an overall decline in pooled prevalence estimates over time, with pneumonia continuing to represent the most frequent infectious complication after stroke [3,4,5]. Beyond their immediate clinical relevance, these infections are observed among patients with longer recorded hospital courses, increased healthcare-related costs, and poorer functional outcomes, further reinforcing their importance from both hospital-management and public health perspectives [6]. Accordingly, the topic remains highly current, as it addresses a persistent source of preventable or modifiable in-hospital morbidity in a patient population already characterized by substantial baseline vulnerability.

The vulnerability of neurological ICU patients to HAIs reflects the combined effects of severe acute brain injury and intensive supportive care. This focus is clinically relevant because the most severe stroke patients often require advanced organ support, including endotracheal intubation and mechanical ventilation, and these patients represent a subgroup with particularly high mortality and complication burden [7,8]. Neurocritical illness has been linked to brain injury-induced immune modulation, while impaired consciousness, dysphagia, aspiration risk, prolonged immobilization, mechanical ventilation, urinary catheterization, vascular access devices, and extended hospital stay all increase susceptibility to infection [9]. Among these factors, post-stroke dysphagia is of particular importance, as it affects more than half of patients in the acute phase and is strongly associated with aspiration pneumonia, dehydration, malnutrition, poor functional outcome, and mortality [4,10,11]. In parallel, the Pneumonia in Stroke Consensus Group has emphasized the need for standardized terminology and operational diagnostic criteria for stroke-associated pneumonia (SAP), a step that is highly relevant when interpreting retrospective hospital datasets and comparing local surveillance findings with the published literature [12]. These considerations are especially important in studies focused on severe or fatal stroke, where infectious complications may represent both markers of critical illness and potential contributors to an unfavorable clinical course.

Within neurocritical care, the predominant infectious complications typically include lower respiratory tract infections, urinary tract infections, bloodstream infections, and device-associated infections, all of which may further complicate the course of severely ill patients [9,13]. This infection-focused perspective is pertinent for Microorganisms and for the Special Issue “Infectious Disease Surveillance in Romania: Third Edition”, particularly because descriptive hospital-based data can help identify documentation gaps and infection-monitoring priorities in the Romanian setting. Recent Romanian data indicate that HAIs caused by multidrug-resistant organisms remain a persistent challenge in acute-care hospitals, particularly in intensive care units, where carbapenem-resistant *Acinetobacter baumannii* and resistant *Klebsiella* spp. continue to play an important epidemiological role [14]. In addition, a recent Romanian neurosurgical study highlighted the clinical value of local surveillance relevant analyses, including the prominent contribution of *Acinetobacter* spp. to hospital-associated infections in a high-risk neurological hospital population [15]. Taken together, these findings support the relevance and timeliness of further hospital-based analyses capable of generating locally meaningful descriptive data on infectious complications and documentation needs.

Despite the growing relevance of HAI surveillance in Romania, data specifically focused on deceased stroke patients admitted to a neurological intensive care unit remain limited. This represents an insufficiently explored but clinically important area at the intersection of stroke care, neurocritical care, hospital epidemiology, and public health microbiology. The justification for the present study is therefore threefold. First, the topic is important because infectious complications may substantially worsen the clinical trajectory of severely ill stroke patients. Second, it is timely because it responds to a continuing need for more granular surveillance-relevant hospital data in Romania, particularly in the context of increasing concern regarding healthcare-associated infections and antimicrobial resistance. Third, the study adds an element of innovation by focusing on a narrowly defined and clinically severe cohort—deceased stroke patients admitted to a neurological intensive care unit—while using a retrospective descriptive dataset derived from a six-year observation window and focused literature contextualization to interpret the local findings within the broader scientific literature. Rather than claiming absolute novelty, the study addresses a domain that remains insufficiently characterized in the Romanian setting and seeks to provide data with direct relevance for hospital-based infection documentation, monitoring, and prevention practice.

Against this background, we hypothesized that healthcare-associated infections represent frequent and clinically relevant documented complications among deceased stroke patients admitted to a neurological intensive care unit, and that the available hospital database could provide an aggregate descriptive profile of these infections within the six-year observation window. Accordingly, the present study was designed to evaluate the burden and profile of healthcare-associated infections among deceased patients admitted with stroke to the Neurological Intensive Care Unit of the Brașov County Emergency Clinical Hospital over a six-year observation period, while exploring the temporal dimension of the available data where supported by database structure and completeness. More specifically, the study aimed to describe the occurrence and distribution of infectious complications in this high-risk cohort, to characterize the demographic and clinical profile of the study population, to assess selected parameters of in-hospital evolution, to describe the aggregate temporal dimension of the available dataset where supported by data structure and completeness, to report available microbiological information associated with documented infections, and to contextualize the local findings through focused literature contextualization addressing post-stroke infection, neurocritical care-associated infections, microbiological documentation, and antimicrobial-resistance context [3,10,14].

## 2. Materials and Methods

### 2.1. Study Design

This study was designed as a retrospective descriptive, single-center hospital-based analysis with focused literature contextualization. The study was not designed to identify predictors of mortality, estimate excess infection risk, or establish causal relationships between documented infectious complications and death. The observational component aimed to characterize the burden and profile of documented healthcare-associated infections among deceased stroke patients admitted to a neurological intensive care unit over a six-year observation period and to explore the temporal dimension of the available dataset where feasible. The literature contextualization component was used to interpret the local descriptive findings in relation to published evidence on post-stroke infection, neurocritical care-associated infections, hospital-based infection documentation, and microbiological data limitations. The reporting of the observational component was structured in accordance with the principles of the STROBE Statement for observational studies [16].

### 2.2. Study Setting and Period

The study was conducted in the Neurological Intensive Care Unit of the Brașov County Emergency Clinical Hospital, Romania. The retrospective study covered a six-year period, from 1 January 2019 to 31 December 2024. The analysis was based on routinely collected hospital data from patients admitted with stroke and who died during the index hospitalization. The institutional setting was considered appropriate for hospital-based descriptive analysis because neurological intensive care populations are exposed to a high burden of systemic and device-associated infections, including respiratory, urinary tract, bloodstream, and other hospital-acquired infectious complications [9,13].

### 2.3. Study Population

The study population included adult patients admitted to the Neurological Intensive Care Unit with a diagnosis of stroke and who died during hospitalization. For the purposes of cohort definition, stroke included all adult patients with a documented diagnosis of acute stroke recorded in the study database, including ischemic stroke and hemorrhagic stroke, according to the available clinical documentation. Eligible cases were identified from the available clinical database and were screened for inclusion according to predefined criteria.

The inclusion criteria were: age ≥ 18 years; admission to the Neurological Intensive Care Unit; a documented diagnosis of stroke; and in-hospital death during the same admission episode.

The exclusion criteria were: duplicate records; cases with insufficient documentation to support the diagnosis of stroke; records with unresolved transfer status or unclear hospitalization trajectory; and cases with major missing data affecting core study variables, particularly those required for cohort definition or for the description of infectious complications and hospital course.

Given the retrospective design and the descriptive purpose of the study, the final cohort was defined pragmatically after data cleaning and consistency checks of the available hospital records [16]. Because the working database was not originally structured as a prospectively curated research registry, cohort selection was based on pragmatic screening for eligibility, in-hospital mortality, and record consistency rather than on a fully itemized exclusion cascade. Accordingly, the cohort-selection flow is reported in aggregate form, distinguishing records available for screening, records not retained after eligibility and record-completeness assessment, and the final deceased-patient cohort included in the descriptive analysis.

Cohort selection was performed from the available working clinical database, which served as the retrospective screening source for case identification, in-hospital mortality filtering, and eligibility assessment. No survivor control group or broader stroke ICU comparison cohort was included. The study therefore describes the infection profile of the fatal stroke ICU subgroup only and was not designed to estimate excess infection risk among deceased patients compared with survivors.

### 2.4. Data Sources and Variable Definition

Clinical and epidemiological data were extracted from the institutional database available for the study period. For analytical clarity, study variables were grouped into four predefined domains.

The first domain included baseline clinical characteristics, namely sex, age, stroke type, selected comorbidities, and relevant neurological antecedents where documented.

The second domain comprised variables related to in-hospital evolution, including length of hospitalization, survival interval during admission, and other available indicators of clinical severity or hospital course.

The third domain included infectious complications of interest, such as pneumonia, urinary tract infection, sepsis, pressure sore-related infection or superinfection, and other documented healthcare-associated infectious events. Because post-stroke pneumonia represents one of the most relevant infectious complications in acute stroke populations, the interpretation of respiratory infection data was conceptually aligned with the terminology proposed by the Pneumonia in Stroke Consensus Group for stroke-associated pneumonia [12].

The fourth domain included microbiological data, where available, namely identified etiological agents and other microbiology-related information considered sufficiently complete and interpretable for descriptive reporting. Microbiological information, where available, was obtained from routinely recorded laboratory and clinical documentation linked to the index hospitalization.

### 2.5. Definition of Healthcare-Associated Infectious Events

For the purposes of this study, healthcare-associated infectious events were defined according to the documentation available in the clinical records and institutional database, with classification focused on the main infectious syndromes captured in routine care. Given the retrospective descriptive nature of the analysis, infectious events were categorized according to documented clinical coding and wording rather than re-adjudicated prospectively.

Where possible, infection categories were interpreted in relation to established healthcare-associated infection surveillance concepts, including CDC/ECDC-oriented terminology. Respiratory infectious complications were also interpreted in light of the literature on stroke-associated pneumonia, which recommends standardized terminology and modified Centers for Disease Control and Prevention-based operational criteria for lower respiratory tract infections occurring after stroke [12]. However, because the source database consisted of routinely recorded retrospective clinical documentation, infectious events could not be fully re-adjudicated according to formal CDC or ECDC case definitions. Therefore, the analysis reports documented infection categories as recorded in the clinical database, with explicit acknowledgement of their limitations for standardized comparability.

### 2.6. Outcomes

The primary descriptive outcome was the distribution of healthcare-associated infection types among deceased stroke patients admitted to the neurological intensive care unit.

The secondary descriptive outcomes included the aggregate temporal dimension of the available dataset where supported by database structure and completeness, the limited microbiological information associated with documented infections, length of stay and survival-related hospitalization parameters, and the distribution of selected demographic and clinical characteristics of the study cohort [9,13].

### 2.7. Data Cleaning and Preparation

Before analysis, the dataset underwent a structured data-cleaning process intended to improve interpretability and reduce internal inconsistencies. This process included verification of duplicate entries, standardization of categorical variables, correction of obvious formatting irregularities in dates and text fields, and assessment of missingness in core variables. Variables with substantial inconsistency or insufficient completeness were retained only for limited descriptive use or were excluded from specific analyses, depending on data quality. A summary of the completeness and interpretability of the key study variables is provided in Supplementary Table S1.

Because the database was primarily assembled for clinical documentation rather than for prospective research, variable harmonization was considered an essential methodological step prior to statistical analysis [16]. Missing or incomplete data were not imputed; each analysis was performed using the available data for the variable of interest.

### 2.8. Statistical Analysis

Data cleaning, harmonization, and statistical analysis were performed between 1 January 2026 and 31 March 2026, after completion of the final study database for the six-year observation period.

Statistical analysis was performed using Microsoft Excel (Microsoft Corp., Redmond, WA, USA) and IBM SPSS Statistics version 26 (IBM Corp., Armonk, NY, USA), as applicable.

The statistical approach was primarily descriptive. Continuous variables were planned to be summarized using mean and standard deviation or median and interquartile range, depending on distributional characteristics and data completeness. Categorical variables were presented as absolute frequencies and percentages. All comparisons between patients with and without documented infectious complications were descriptive only. No inferential testing was performed, and observed differences should not be interpreted as statistical associations, predictors, or causal relationships.

The main analysis focused on descriptive characterization of the cohort and documented infectious patterns. The temporal dimension of the six-year observation period was explored only to the extent supported by data quality, structure, and completeness, and was ultimately interpreted as an aggregate descriptive dimension rather than as a formal annual trend analysis. The central objective remained descriptive hospital-based reporting using routinely collected clinical data.

### 2.9. Literature Contextualization Approach

In parallel with the observational component, a focused literature contextualization was performed in order to interpret the local findings in relation to published evidence on post-stroke infections, stroke-associated pneumonia, urinary tract infections, healthcare-associated infections in neurocritical care, microbiological documentation, and Romanian data relevant to hospital-associated infections. This component was not designed as a systematic review or meta-analysis and therefore did not require a PRISMA flow diagram. Its purpose was to support interpretation of the descriptive findings generated from the local cohort rather than to provide an independent evidence synthesis.

The literature search was primarily performed in PubMed/MEDLINE, using combinations of terms related to stroke, healthcare-associated infections, neurocritical care, stroke-associated pneumonia, urinary tract infection, antimicrobial resistance, and Romania. Reference selection was guided by thematic relevance, methodological robustness, and applicability to the objectives of the present study. Priority was given to recent peer-reviewed articles, consensus documents, systematic reviews, and studies with direct relevance to neurocritical care and the Romanian healthcare context.

## 3. Results

### 3.1. Cohort Selection and General Characteristics

**Figure 1:**
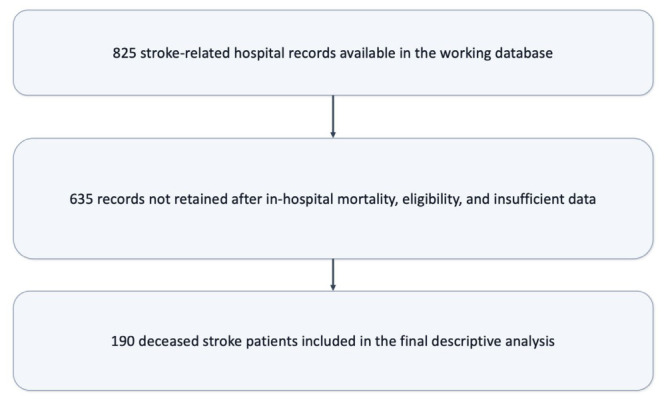
Study Cohort Selection Flowchart. Explanatory note: The flowchart summarizes the aggregate screening and selection of the final study cohort from the stroke-related hospital records available in the working database. Records not retained included cases that did not meet the in-hospital mortality requirement, eligibility criteria, or record-completeness requirements for the present descriptive analysis. Because the source dataset was retrospectively assembled for clinical documentation rather than prospective research tracking, the exclusion cascade is presented in aggregate form.

A total of 825 stroke-related hospital records were available in the working database and were screened for in-hospital mortality and eligibility according to the predefined cohort criteria. After this selection process, 190 deceased patients met the inclusion criteria and were retained in the final descriptive analysis, whereas 635 records were not retained because they did not meet the mortality, eligibility, or record-completeness requirements for the present study (Figure 1). These cases constituted the study cohort of deceased stroke patients admitted to the Neurological Intensive Care Unit.

The baseline clinical characteristics of the study cohort are summarized in Table 1. The available dataset supported descriptive evaluation of the baseline cohort profile, infectious complications, and hospitalization course. However, several variables showed incomplete or heterogeneous recording, and all analyses were therefore performed on available data only, in accordance with the methodological approach outlined above. The completeness and interpretability of key variables are summarized in Supplementary Table S1.

### 3.2. Stroke Profile and Baseline Clinical Characteristics of Deceased Patients

**Figure 2:**
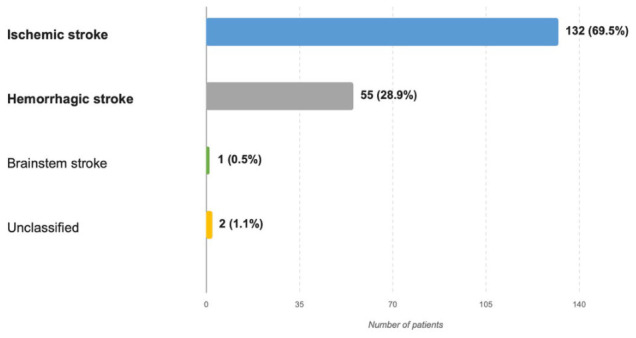
Distribution of Stroke Subtypes in the Deceased Study Cohort. Explanatory note: The figure illustrates the distribution of stroke subtypes within the final cohort of deceased patients admitted to the Neurological Intensive Care Unit. Stroke classification was based on the available coding structure in the working clinical database and included ischemic stroke, hemorrhagic stroke, brainstem stroke, and insufficiently classifiable cases.

Within the final cohort, ischemic stroke was the predominant subtype, accounting for 132 of 190 cases (69.5%), whereas hemorrhagic stroke accounted for 55 cases (28.9%). One patient (0.5%) was coded as having brainstem stroke, while two cases (1.1%) remained insufficiently classifiable on the basis of the available coding structure (Table 1, Figure 2).

Sex distribution was nearly balanced, with a slight predominance of female patients. Overall, 104 patients (54.7%) were female and 85 (44.7%) were male; one additional record (0.5%) contained ambiguous sex coding and could not be classified with certainty (Table 1).

Regarding baseline clinical profile, hypertension was the most frequently documented comorbidity, being recorded in 140 patients (73.7%). Ischemic heart disease and/or previous myocardial infarction were present in 114 cases (60.0%), atrial fibrillation in 62 (32.6%), renal disease in 48 (25.3%), diabetes mellitus in 46 (24.2%), hepatic disease in 34 (17.9%), obesity in 31 (16.3%), previous stroke in 24 (12.6%), and malignancy in 15 cases (7.9%) (Table 1).

The age variable was not sufficiently complete or internally consistent in the available dataset to support reliable descriptive reporting and was therefore not included in the present results.

### 3.3. Burden and Pattern of Healthcare-Associated Infections

Healthcare-associated infectious complications were frequently documented in this cohort of deceased stroke patients. The distribution of main documented infection categories and recorded specifications within these categories is summarized in Table 2. Pneumonia represented the dominant documented infectious complication, being recorded in 100 of 190 patients (52.6%). Within the pneumonia category, 69 cases (36.3% of the total cohort) were recorded without further etiological specification, while 30 cases (15.8%) were documented as COVID-19-associated pneumonia and one case (0.5%) as Pseudomonas-associated pneumonia.

Urinary tract infection was documented in 22 patients (11.6%). Most entries were nonspecific, although one case was explicitly recorded as Proteus-associated urinary tract infection.

Pressure sore-related infection was explicitly recorded in 9 patients (4.7%). In addition, 13 records (6.8%) contained sepsis-related wording in the source dataset. Because this wording was heterogeneous and not supported by sufficient standardized detail for retrospective adjudication, it was retained only as a descriptive coding category and should not be interpreted as confirmed sepsis.

**Figure 3:**
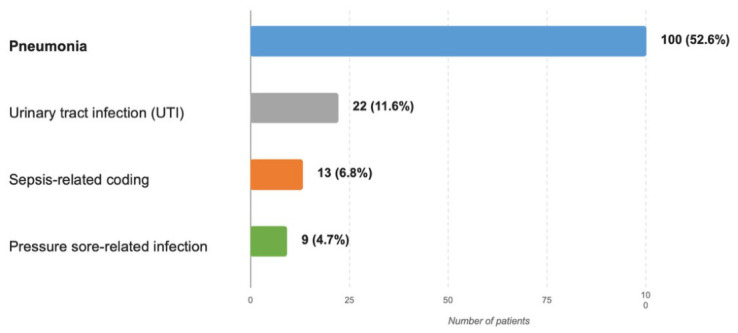
Distribution of Major Healthcare-Associated Infectious Complications in the Study Cohort. Explanatory note: The figure illustrates the distribution of the major documented healthcare-associated infectious complications in the final cohort of deceased stroke patients, including pneumonia, urinary tract infection, pressure sore-related infection, and sepsis-related coding. Frequencies are presented as absolute numbers, with percentages calculated using the full study cohort (*n* = 190) as the denominator.

Taken together, the available data indicate that lower respiratory tract infections represented the principal documented infectious category in this fatal neurological ICU cohort, followed by urinary tract infections, whereas pressure sore-related infection and sepsis-related coding were less frequently recorded (Figure 3).

### 3.4. Temporal Dimension of the Study Period

The study covered a six-year retrospective observation period from which the final deceased-patient cohort was derived. However, the available database did not support sufficiently robust year-by-year stratification to allow valid quantitative reporting of annual case volume, annual infection proportions, or longitudinal microbiological changes across the full study interval. Therefore, the temporal component of the present analysis was interpreted in aggregate form. Accordingly, the six-year observation period should be understood as the retrospective time window from which the final cohort was derived, rather than as the basis for a formal longitudinal or annual trend analysis.

### 3.5. Clinical Evolution and Hospital Course

The hospitalization course of the deceased cohort was characterized by a relatively short, yet clinically heterogeneous, survival interval. Hospital course and survival-related parameters according to documented infectious complications are summarized in Table 3. In the overall cohort, the median in-hospital survival interval was 6 days (interquartile range, 3–10.75 days), while the mean survival interval was 7.9 days, with an observed range from 1 to 46 days.

Descriptive comparison showed longer observed in-hospital survival intervals among patients with documented infectious complications; however, these comparisons were purely descriptive and should not be interpreted as evidence of statistical association or causality.

Patients with documented pneumonia had a median in-hospital survival interval of 7 days, compared with 5 days among those without documented pneumonia. For urinary tract infection, the median interval was 10.5 days in patients with documented infection and 6 days in those without documented urinary infection. Patients with documented pressure sore-related infection had a median interval of 11 days. Records containing sepsis-related coding had a median interval of 20 days.

These findings indicate that documented infectious complications were observed more frequently among patients with longer recorded hospital courses, a pattern that may partly reflect greater time at risk for infection development or documentation. No causal or inferential interpretation should be made from these descriptive comparisons.

## 4. Discussion

The literature contextualization component was used to interpret the descriptive findings of the local cohort rather than to provide an independent systematic evidence synthesis. Accordingly, the discussion is organized around the main cohort findings: predominance of pneumonia, documentation of urinary tract infection and other infectious categories, limited microbiological granularity, and the implications of these findings for hospital-based infection documentation in the Romanian neurocritical care context.

### 4.1. Why Stroke Patients in Neuro-ICU Are Particularly Prone to Healthcare-Associated Infections

The present study highlights a substantial burden of healthcare-associated infections in deceased stroke patients admitted to a neurological intensive care unit, with respiratory infections emerging as the predominant complication. This finding is biologically and clinically plausible. Patients with severe stroke represent a particularly vulnerable population because of the combined effects of the primary neurological insult, impaired consciousness, dysphagia, aspiration risk, prolonged immobilization, exposure to invasive devices, and extended hospitalization [4,9,10,11]. In addition, severe acute brain injury has been associated with stroke-related immune dysregulation, which may further increase susceptibility to infection during the acute and subacute phases of critical illness [9]. This vulnerability is further supported by recent evidence identifying advanced age, dysphagia, impaired consciousness, high stroke severity, diabetes, elevated inflammatory markers, and nasogastric tube use among the principal factors associated with stroke-associated pneumonia in ischemic stroke populations [4]. The present cohort should also be viewed in the context of severe stroke populations requiring high-dependency or intensive care support, in whom respiratory compromise, depressed consciousness, and the need for intubation or mechanical ventilation further amplify the risk of infectious complications and adverse outcome [7,8].

In the Romanian setting, this vulnerability may be further amplified by the limited availability of specialized post-stroke neurorehabilitation pathways and by restricted access to early rehabilitation after the acute phase. Delayed initiation of mobilization and recovery-oriented care may prolong immobilization, device exposure, and dependence on high-intensity hospital care, thereby potentially increasing susceptibility to healthcare-associated infections and contributing to an unfavorable clinical course [17].

These mechanisms are especially relevant in neurocritical care settings, where lower respiratory tract infections, urinary tract infections, bloodstream infections, and device-associated infections are well recognized complications in patients requiring intensive supportive care [9,13]. The predominance of pneumonia in the present cohort is therefore consistent with the broader neurocritical care literature and with the expected vulnerability of severe stroke patients to respiratory infectious complications.

Although the study covered a six-year retrospective observation period, the available database did not permit sufficiently granular year-by-year stratification for formal temporal trend analysis. The temporal dimension should therefore be interpreted as an aggregate descriptive feature of the dataset rather than as a longitudinal comparison across individual calendar years. Consequently, the results support only a descriptive interpretation of the infectious burden in the final deceased-patient cohort, without implying formal temporal persistence or annual trend.

### 4.2. Stroke-Associated Pneumonia as the Dominant Infectious Complication

The most prominent finding of the present study is the clear predominance of pneumonia, which was documented in more than half of the deceased cohort. This observation is in line with the literature indicating that pneumonia is the most frequent infectious complication after stroke and one of the most prognostically important [2,3,5,18,19]. Westendorp et al. showed that post-stroke infections complicate a substantial proportion of acute stroke cases and that pneumonia, in particular, is associated with increased mortality [2]. More recent pooled evidence has confirmed that pneumonia remains the dominant infectious complication despite a gradual decline in overall post-stroke infection prevalence over time [3].

In our cohort, the predominance of pneumonia is particularly meaningful because the population studied consisted exclusively of deceased patients admitted to a neurological intensive care unit. In such patients, the convergence of severe neurological injury, impaired airway protection, dysphagia, aspiration risk, immobility, and intensive care exposure creates the ideal context for respiratory infectious complications [9,10]. This is also consistent with the conceptual framework of stroke-associated pneumonia proposed by the Pneumonia in Stroke Consensus Group, which emphasized the need for standardized terminology and operational diagnostic criteria for lower respiratory tract infections complicating stroke, including the distinction between probable and definite SAP according to modified CDC-based criteria [12]. Although our retrospective database did not permit formal adjudication of SAP according to modified CDC-based criteria, the high frequency of documented pneumonia indicates that respiratory infection was the dominant recorded infectious category in this fatal cohort. In addition, subsequent PISCES recommendations have emphasized the importance of standardized empirical antibiotic strategies for pneumonia complicating stroke, taking into account timing from stroke onset and likely microbiological profiles [20].

In the present cohort, patients with documented pneumonia had a higher median in-hospital survival interval than those without documented pneumonia. This difference was descriptive only and should not be interpreted as evidence that pneumonia caused a longer hospital course. A more cautious interpretation is that patients with longer recorded hospitalization had more time at risk for pneumonia development or documentation. In this setting, pneumonia should therefore be viewed as a frequent documented complication within a severe fatal hospital course, rather than as a proven determinant of hospitalization duration or death. This interpretation is compatible with previous literature showing that post-stroke infections are associated with prolonged hospitalization, higher healthcare-related costs, and poorer outcomes [6]. Recent meta-analytic evidence further supports the prognostic relevance of stroke-associated pneumonia, showing increased in-hospital mortality, increased mortality at 1 to 3 months, and a significantly higher risk of poor functional outcome in stroke patients who develop pneumonia [19].

Recent prospective multicenter data also suggest that pneumonia fulfilling modified CDC-based criteria may identify the subgroup with the greatest prognostic relevance. In such cohorts, stroke-associated pneumonia defined according to mCDC criteria, rather than other clinically suspected pneumonias, has been associated with poor outcome, while antibiotic initiation in practice may still be driven more by isolated clinical signs such as fever than by strict adherence to predefined criteria [18].

### 4.3. Urinary Tract Infections and Device-Associated Complications

Urinary tract infection represented the second most frequent documented infectious complication in the present cohort. Although substantially less frequent than pneumonia, it remains clinically relevant, particularly in the context of critically ill stroke patients, in whom urinary catheterization, immobility, neurological impairment, and prolonged hospitalization may all contribute to increased risk [9]. In the descriptive hospital-course analysis, patients with documented urinary tract infection had a higher median in-hospital survival interval than those without documented urinary infection. As with pneumonia, this finding should be interpreted cautiously and may primarily reflect greater time at risk for infection development or documentation among patients with longer recorded hospital courses. This cautious interpretation is compatible with prior stroke-focused literature indicating that post-stroke urinary tract infection is closely linked to urinary catheterization, increasing age, and greater post-stroke disability, and has been reported in association with longer hospital stays and increased healthcare costs [21,22].

This observation is consistent with the broader post-stroke literature, in which urinary tract infection is recognized as one of the most common infectious complications after stroke, although generally less consequential than pneumonia from a mortality standpoint [2,3]. In the ICU context, UTI also has relevance as a marker of device exposure and hospital-acquired morbidity. Even when microbiological detail is limited, the presence of documented urinary infection in a cohort of deceased neurocritical care patients should not be minimized, because it may reflect broader issues related to catheter management, documentation quality, and infection prevention practice. This interpretation is also supported by stroke-focused review data showing that post-stroke urinary tract infection is consistently associated with longer recorded hospital courses, discharge to institutional care, and greater healthcare utilization, even when its independent association with long-term mortality appears less consistent than that of pneumonia [23]. Importantly, prior reviews have emphasized that stroke patients represent a specific high-risk population for urinary tract infection because of bladder dysfunction, immunological changes, and the frequent use of indwelling urinary catheters, making catheter stewardship a potentially relevant preventive target in stroke care [21].

Pressure sore-related infection and sepsis-related coding were less frequent in absolute terms and should be interpreted with particular caution because of the small numbers and heterogeneous documentation. Their presence nevertheless indicates that the documented infectious profile in this cohort was not limited to respiratory complications alone.

### 4.4. Microbiological Documentation and Antimicrobial Resistance Context

The microbiological component of the present study was limited and should be interpreted only as contextual and exploratory. The available dataset contained isolated etiological specifications, including COVID-19-associated pneumonia, Pseudomonas-associated pneumonia, and Proteus-associated urinary tract infection, but it did not allow pathogen-distribution analysis, antimicrobial susceptibility assessment, or antimicrobial resistance profiling. Therefore, the present findings should not be interpreted as microbiological surveillance results derived from the cohort.

This limitation is particularly relevant in stroke-associated pneumonia, where the likely microbiological spectrum may vary according to the timing of onset after stroke and the healthcare setting. Consensus recommendations from the PISCES group suggest that early pneumonia complicating stroke should generally be approached as community-acquired pneumonia, whereas later presentations may require broader consideration of hospital-associated organisms, including Gram-negative pathogens depending on clinical risk factors [20]. Similarly, studies using modified CDC-based criteria have emphasized that clinically suspected pneumonia in stroke patients may not always correspond to more strictly defined stroke-associated pneumonia, with implications for antibiotic initiation and stewardship [18].

References to antimicrobial resistance and Romanian microbiological ecology are therefore used only as contextual background and as justification for more standardized future data collection, not as conclusions generated by the present dataset. Recent Romanian studies have highlighted the burden of multidrug-resistant pathogens in acute-care and neurosurgical hospital settings, particularly involving Gram-negative organisms such as *Acinetobacter baumannii* and resistant *Klebsiella* spp. [14,15]. In this context, the main implication of the present study is the need for better linkage between clinical infection documentation, microbiological isolates, antimicrobial susceptibility data, and infection prevention measures in future neurocritical care datasets.

### 4.5. Romanian Relevance and Hospital-Based Infection Documentation

The Romanian relevance of the study lies primarily in its hospital-based description of documented infectious complications in a severe and narrowly defined neurocritical care cohort. Rather than representing formal national surveillance evidence, the study provides local descriptive data that may help identify documentation gaps and infection domains requiring closer monitoring in Romanian neurological ICU practice.

This relevance can be considered from several complementary perspectives. First, it addresses a topic for which dedicated hospital-based data remain limited, namely healthcare-associated infections in deceased stroke patients requiring neurocritical care. Second, it contributes to awareness regarding the infectious burden that may complicate severe stroke management in intensive care settings. Third, it highlights the need for more standardized clinical documentation and better compatibility between routine hospital records and infection-monitoring requirements, especially when hospital-derived databases are later used for retrospective epidemiological analysis. From this perspective, the present study also underscores the need for stroke-unit and neuro-ICU datasets that are more tightly aligned with structured infection definitions and documentation criteria, particularly for pneumonia and device-associated complications [18,20,23].

When compared with broader post-stroke cohorts, the infection burden observed in the present study appears higher, as expected for a cohort restricted to deceased neurological ICU patients. Previous meta-analytic data reported post-stroke infection in approximately 30% of acute stroke patients, with pneumonia and urinary tract infection each occurring in around 10% of cases, whereas in the present fatal ICU cohort pneumonia was documented in 52.6% and urinary tract infection in 11.6% of patients [2]. This comparison suggests that the respiratory infectious burden observed in our cohort is consistent with the expected enrichment of complications in a more severe fatal neurocritical care population, rather than being directly generalizable to the wider stroke population.

The study also has practical implications for local infection prevention priorities. The predominance of pneumonia suggests that aspiration risk, dysphagia assessment, respiratory surveillance, and early preventive measures remain central concerns in severe stroke populations [10,12]. The presence of urinary tract infections and other infectious complications further supports continued attention to catheter-related risk, immobilization-related morbidity, and broader ICU infection prevention protocols. In this sense, the value of the present study is not limited to description alone; it also lies in helping define which infectious domains deserve the greatest preventive attention in Romanian neurological ICU practice. From a broader neurocritical care perspective, systematic review data suggest that meaningful reductions in ICU-acquired infections are most likely to be achieved through collaborative, multidisciplinary, and bundle-based prevention strategies rather than isolated measures alone [24].

### 4.6. Strengths and Limitations

This study has several strengths. It focuses on a highly specific and clinically important cohort, namely deceased stroke patients admitted to a neurological intensive care unit, a population in whom documented infectious complications are likely to be particularly relevant but remain insufficiently characterized in the Romanian setting. The study also provides a structured hospital-based descriptive profile supported by clearly defined cohort criteria, transparent reporting of data completeness, and focused literature contextualization. In this way, the manuscript places local findings within the broader framework of neurocritical care infection, stroke-associated pneumonia, urinary tract infection, and infection-documentation challenges.

At the same time, several limitations must be acknowledged. First, the study is retrospective and single-center in design, which limits external generalizability. Second, the source database was assembled for routine clinical documentation rather than as a prospectively curated research registry. This affected the granularity and consistency of some variables and required a pragmatic rather than fully itemized approach to cohort selection. Third, although the study covered a six-year retrospective observation period, the available clinical database did not support a robust year-by-year stratified analysis. The temporal component should therefore be interpreted as an aggregate descriptive dimension of the dataset rather than as a formal longitudinal trend analysis.

Additional limitations concern data completeness and standardization. The age variable was insufficiently complete and internally inconsistent for reliable inclusion in the present analysis. Some infectious complications, particularly sepsis-related coding and other non-respiratory events, were recorded heterogeneously and could therefore only be interpreted descriptively. Microbiological information was available only to a limited extent and did not permit a detailed pathogen-level or resistance-level analysis. This is particularly relevant because recent studies emphasize that microbiological characterization and antibiotic stewardship remain insufficiently standardized in pneumonia complicating stroke [18,20]. Finally, because the cohort was restricted to deceased patients, the findings should not be interpreted as representative of the entire stroke ICU population, but rather of its most severe and clinically unfavorable segment.

The restriction to deceased patients may also be viewed as a focused feature of the study, because it captures the subgroup with maximum clinical severity and unfavorable outcome. However, this should not be interpreted as improving generalizability. Rather, it emphasizes the need for future studies including both deceased and surviving stroke ICU patients, prospectively structured infection definitions, standardized microbiological linkage, and predefined analytical strategies capable of supporting comparative and risk-modeling analyses.

## 5. Conclusions

Deceased stroke patients admitted to a neurological intensive care unit represent a highly vulnerable subgroup in whom documented healthcare-associated infections were frequent in the available retrospective dataset. In the present cohort, respiratory infections—particularly pneumonia—represented the dominant documented infectious category, while urinary tract infection, pressure sore-related infection, and sepsis-related coding were less frequently recorded. Observed hospital-course differences according to infection documentation were descriptive only and should not be interpreted as evidence of statistical association or causality.

Although the study covered a six-year observation period, the temporal component remained aggregate and should not be interpreted as formal annual trend analysis. The findings therefore provide a descriptive hospital-based profile of documented infectious complications in a fatal stroke ICU cohort, rather than generalizable surveillance or microbiological evidence.

These findings support the need for more standardized clinical documentation, clearer infection coding, and better linkage between clinical and microbiological data in neurocritical care settings. Future studies should include both deceased and surviving stroke ICU patients, prospectively structured infection definitions, standardized microbiological linkage, and predefined analytical strategies capable of supporting comparative analyses and risk modeling.

## Figures and Tables

**Table 1:** Baseline Clinical Characteristics of the Study Cohort of Deceased Stroke Patients Admitted to the Neurological Intensive Care Unit.

Characteristic	Value
**Total study cohort, *n***	**190**
**Sex, *n* (%)**	
Female	104 (54.7)
Male	85 (44.7)
Ambiguous/unclassified	1 (0.5)
**Stroke subtype, *n* (%)**	
Ischemic stroke	132 (69.5)
Hemorrhagic stroke	55 (28.9)
Brainstem stroke	1 (0.5)
Insufficiently classifiable	2 (1.1)
**Documented comorbidities, *n* (%)**	
Hypertension	140 (73.7)
Ischemic heart disease and/or prior myocardial infarction	114 (60.0)
Atrial fibrillation	62 (32.6)
Renal disease	48 (25.3)
Diabetes mellitus	46 (24.2)
Hepatic disease	34 (17.9)
Obesity	31 (16.3)
Previous stroke	24 (12.6)
Malignancy	15 (7.9)

Explanatory notes: Percentages are reported using the full study cohort (*n* = 190) as the denominator and are rounded to one decimal place. Comorbidities were documented from the available clinical records and were not mutually exclusive. The age variable was not included in this table because it was insufficiently complete and internally inconsistent in the working database.

**Table 2:** Distribution of Healthcare-Associated Infectious Complications in the Study Cohort.

Documented Infectious Complication	*n*	%
**Main documented infection categories**		
Pneumonia, overall	100	52.6
Urinary tract infection, overall	22	11.6
Pressure sore-related infection	9	4.7
Sepsis-related coding	13	6.8
**Documented specifications within infection categories**		
Pneumonia without further etiological specification	69	36.3
COVID-19-associated pneumonia	30	15.8
*Pseudomonas*-associated pneumonia	1	0.5
*Proteus*-associated urinary tract infection	1	0.5

Explanatory notes: Percentages are reported using the full study cohort (*n* = 190) as the denominator and are rounded to one decimal place. Main infection categories and documented specifications are presented separately. Specifications represent recorded etiological or contextual details within broader infection categories and should not be summed with parent categories. Sepsis-related coding indicates non-standardized sepsis-related wording in the source dataset and does not represent retrospectively adjudicated sepsis.

**Table 3:** Hospital Course and Survival-Related Parameters According to Documented Infectious Complications.

Group	*n*	Median Survival Interval (Days)	IQR (Days)	Mean Survival Interval (Days)
**Overall cohort**	190	6.0	3.0–10.75	7.9
**Pneumonia**				
Yes	100	7.0	3.0–12.0	9.0
No	90	5.0	3.0–8.75	6.7
**UTI**				
Yes	22	10.5	5.0–20.0	15.1
No	168	6.0	3.0–9.0	7.0
**Pressure sore-related infection**				
Yes	9	11.0	6.0–14.0	12.8
No	181	6.0	3.0–10.0	7.7
**Sepsis-related coding**				
Yes	13	20.0	6.0–23.0	16.5
No	177	6.0	3.0–10.0	7.3

Explanatory notes: Survival interval refers to the in-hospital survival interval, expressed in days, and was derived from the working clinical database for the final deceased-patient cohort (*n* = 190). Interquartile range (IQR) is reported as the 25th to 75th percentile. Infectious complication groups were defined according to the documented presence or absence of pneumonia, urinary tract infection (UTI), pressure sore-related infection, or sepsis-related coding in the source dataset. Mean values are rounded to one decimal place. These comparisons are descriptive only and should not be interpreted as evidence of statistical association or causality.

## Data Availability

The data analyzed in this study were derived from routinely collected institutional clinical records. Due to institutional and ethical restrictions, the dataset is not publicly available, but may be made available from the corresponding author on reasonable request and with permission of the hosting institution.

## Supplementary Materials

The following supporting information can be downloaded at: https://www.mdpi.com/article/10.3390/microorganisms14051062/s1, Table S1: Completeness and Interpretability of Key Study Variables.

## Abbreviations

The following abbreviations are used in this manuscript:

CDC	Centers for Disease Control and Prevention
ECDC	European Centre for Disease Prevention and Control
HAI/HAIs	Healthcare-associated infection/healthcare-associated infections
ICU	Intensive Care Unit
IQR	Interquartile range
mCDC	Modified Centers for Disease Control and Prevention
SAP	Stroke-associated pneumonia
STROBE	Strengthening the Reporting of Observational Studies in Epidemiology
UTI	Urinary tract infection
