# Supplementary material for: Healthcare-Associated Infections in Deceased Stroke Patients in a Romanian Neurological ICU: A Retrospective Descriptive Study
Source: Microorganisms. 2026 May 8;14(5):1062. doi: 10.3390/microorganisms14051062 (PMC13209331; doi:10.3390/microorganisms14051062)
Supplement: Supplementary file 1 [file microorganisms-14-01062-s001.zip › microorganisms-4270525-supplementary.docx]

**Supplementary Table S1.** Completeness and Interpretability of Key Study Variables.

| **Variable domain** | **Variable** | **Availability / interpretability** | **Use in analysis** | **Comment** |
| --- | --- | --- | --- | --- |
| Cohort definition | In-hospital death | Sufficient for cohort selection | Included | Used to define the final deceased-patient cohort. |
| Cohort definition | Admission to Neurological Intensive Care Unit | Sufficient for cohort selection | Included | Used as part of the predefined eligibility criteria. |
| Cohort definition | Documented stroke diagnosis | Sufficient for cohort selection | Included | Used to identify eligible stroke-related hospital records. |
| Baseline profile | Sex | Sufficiently complete | Included | One record contained ambiguous / unclassified sex coding. |
| Baseline profile | Stroke subtype | Sufficiently complete | Included | Two records were insufficiently classifiable on the basis of the available coding structure. |
| Baseline profile | Age | Incomplete / internally inconsistent | Excluded from descriptive reporting | Not included in the final descriptive results because the variable was not sufficiently complete and internally consistent. |
| Baseline profile | Documented comorbidities | Available as recorded clinical documentation | Included descriptively | Comorbidities were not mutually exclusive and were reported as documented in the source dataset. |
| Infection profile | Pneumonia | Available as documented coding | Included | Reported according to documented clinical coding; not re-adjudicated according to formal CDC/ECDC criteria. |
| Infection profile | Urinary tract infection | Available as documented coding | Included | Reported according to documented clinical coding; etiological specification was limited. |
| Infection profile | Pressure sore-related infection | Available as documented coding | Included descriptively | Interpreted as a documented infectious complication; not prospectively adjudicated. |
| Infection profile | Sepsis-related coding | Heterogeneous wording in the source dataset | Included with caution | Retained only as a descriptive coding category; not equivalent to retrospectively adjudicated sepsis. |
| Microbiology | Pathogen identification | Limited | Descriptive only | Not suitable for pathogen-level epidemiological analysis. |
| Microbiology | Antimicrobial resistance profile | Insufficient / not systematically available | Not analyzed | The dataset did not support antimicrobial resistance profiling. |
| Hospital course | In-hospital survival interval | Sufficient for descriptive analysis | Included | Used to describe hospital-course parameters in the final deceased-patient cohort. |
| Temporal dimension | Calendar-year stratification | Insufficiently robust for formal annual analysis | Aggregate interpretation only | The six-year period was interpreted as an observation window, not as the basis for formal year-by-year trend analysis. |
